# TLR7 promotes chronic airway disease in RSV-infected mice

**DOI:** 10.3389/fimmu.2023.1240552

**Published:** 2023-09-14

**Authors:** Mark A. Miles, Stella Liong, Felicia Liong, Madison Coward-Smith, Gemma S. Trollope, Osezua Oseghale, Jonathan R. Erlich, Robert D. Brooks, Jessica M. Logan, Shane Hickey, Hao Wang, Steven Bozinovski, John J. O’Leary, Doug A. Brooks, Stavros Selemidis

**Affiliations:** ^1^ Centre for Respiratory Science and Health, School of Health and Biomedical Sciences, RMIT University, Bundoora, VIC, Australia; ^2^ Clinical and Health Sciences, University of South Australia, Adelaide, SA, Australia; ^3^ Discipline of Histopathology, School of Medicine, Trinity Translational Medicine Institute (TTMI), Trinity College Dublin, Dublin, Ireland; ^4^ Sir Patrick Dun’s Laboratory, Central Pathology Laboratory, St James’s Hospital, Dublin, Ireland

**Keywords:** toll-like receptor 7, respiratory syncytial virus, inflammation, airway hyperreactivity, viral infection

## Abstract

Respiratory syncytial virus (RSV) commonly infects the upper respiratory tract (URT) of humans, manifesting with mild cold or flu-like symptoms. However, in infants and the elderly, severe disease of the lower respiratory tract (LRT) often occurs and can develop into chronic airway disease. A better understanding of how an acute RSV infection transitions to a LRT chronic inflammatory disease is critically important to improve patient care and long-term health outcomes. To model acute and chronic phases of the disease, we infected wild-type C57BL/6 and toll-like receptor 7 knockout (TLR7 KO) mice with RSV and temporally assessed nasal, airway and lung inflammation for up to 42 days post-infection. We show that TLR7 reduced viral titers in the URT during acute infection but promoted pronounced pathogenic and chronic airway inflammation and hyperreactivity in the LRT. This study defines a hitherto unappreciated molecular mechanism of lower respiratory pathogenesis to RSV, highlighting the potential of TLR7 modulation to constrain RSV pathology to the URT.

## 1 Introduction

Respiratory syncytial virus (RSV) causes widespread global infection, with most children under the age of two being infected. While infection typically manifests in the upper respiratory tract (URT) and presents as a mild cold, it is estimated that a tenth of the 34 million global cases annually progress to a lower respiratory tract (LRT) infection (LRTI) leading to bronchiolitis and even hospitalization, particularly in the young children and elderly (1). Indeed, nearly a quarter of LRTI-related deaths in young children are associated with RSV, and this disease is the leading cause of hospitalization in infants (2). Although high-risk groups such as pre-term infants or those with existing respiratory or congenital heart disease are more likely to be hospitalized, most children experiencing severe RSV infection have been previously healthy; highlighting the indiscriminate nature of the disease progression (3). Frequent reinfection is also common in older children and adults, highlighting a susceptibility issue and lack of established long-term immunity after infection (4). Encouragingly, two RSV vaccines were recently FDA approved for use in older adults (5, 6), although effective therapeutic interventions for all patients with an RSV infection is still limited (7).

Infants who develop severe bronchiolitis due to RSV infection are at risk of developing recurrent wheezing, asthma and/or allergic sensitization (8, 9), emphasizing the potential long-term disease burden caused by this virus. The increased susceptibility of infected individuals to other respiratory disease (e.g. asthma), could be due to heightened RSV-driven airway reactivity and/or tissue injury, which has resulted from RSV infection during infant lung development (10). Pulmonary and mucosal immunoregulatory responses of immune and epithelial cells to RSV infection are also important determinants of disease severity and exacerbation in both children and adults (11). This is likely due to the developing immune landscape in infants, which is temporally shaped by its response to environmental and pathological challenges in early life (12). RSV infection also tends to create an age-dependent type-2 skewed immunological environment in infants that often results in a suboptimal antiviral response and chronic inflammation (13). Furthermore, adults (stronger Th1 immune bias) are more likely to succumb to RSV infection when neutrophil numbers in the mucosa are high at the time of inoculation (14). Mouse studies using C57BL/6 mice (representing a Th1 skewed animal model) recapitulating the human challenge trials described above revealed neutrophilic inflammation promoted an exacerbated cytotoxic CD8+ T cell response that underpins the long-term respiratory disease, including airway hyperactivity (14). An important balance of host Th1, Th2 and Th17 antiviral and proinflammatory immune responses is therefore required to ensure efficient viral clearance and to avoid uncontrolled excessive inflammation (11), which can have damaging effects on the airways and can also enhance viral pathogenicity.

RSV is a single-stranded RNA (ssRNA) virus that enters host cells via a pH-independent fusion mechanism, although proteolytic cleavage of the Fusion protein by host furin was reported in a low pH-dependent manner in early endosomes, implicating the involvement of the endocytic pathway in RSV biology (15, 16). However, RSV infection of certain cell types occurs directly into the cytosol and is independent of endosomal compartments (17). Pattern recognition receptors (PRRs) such as toll-like receptors (TLRs), some of which reside within the endosome, initiate important innate responses to infection upon sensing of pathogen-associated molecular patterns (18), but as RSV can enter directly into the cytosol this may limit detection by these TLRs until later in the infection process. TLR7 senses endosomally localized ssRNA and its activation triggers downstream induction of transcription factor genes, such as interferon regulatory factors (IRFs) or nuclear factor-κB (NFκB), to mediate host antiviral responses that are critical for establishing an appropriate immune response. The sensing of ssRNA viruses, such as influenza A (IAV), SARS-CoV2 and RSV, is dependent on a high level of TLR7 being expressed in plasmacytoid dendritic cells (pDCs), patrolling monocytes, macrophages and B cells, and is necessary to modulate various arms of the innate and adaptive immune system (19). Retinoic acid-inducible gene I (RIG-I)-like receptors (RLRs) are another family of PRRs that also potently induce type I IFN responses upon cytosolic sensing of ssRNA (20), and these are important for early antiviral responses to RSV infection (21). Due to the potential of RSV to be sensed in either the cytosol by RLRs and/or the endosome by TLR7/8, both systems may contribute to hyperinflammation in response to RSV infection either individually, sequentially or simultaneously.

TLR7 has a potential protective role during the acute phases of viral infection (22–25) by driving an early Th1 response in the lungs to evoke an antiviral response. However, a hyperinflammatory “cytokine storm” setting within the airways and lungs could arise if TLR7-mediated inflammatory responses are not appropriately resolved following viral clearance. This has been recently shown for SARS-CoV2 infection where pDC-dependent TLR7 activation by the virus drives type I IFN signaling and mediates both transcriptional and epigenetic alterations in macrophages to favor their hyperactivation in patients with severe COVID-19 (26). Similarly, during severe IAV infection, the pharmacological suppression of TLR7 reduced monocyte- and neutrophil-driven lung inflammation, which restricted the resultant downstream pathology (27). The establishment of an immunopathological niche following improper immune regulation and excess inflammation can potentiate viral pathogenicity and exacerbate chronic airway inflammatory disease (28). Fewer studies have investigated the role of TLR7 in RSV infection, which is the focus herein. While these studies have identified enhanced mucus production in the airways (29) or delayed innate pDC immune responses that impact viral clearance (30) in TLR7-deficient mice, the ability of TLR7 to potentiate RSV-induced chronic respiratory disease has not been previously investigated. Interestingly, RSV transcripts were detected in the lungs of mice several months following infection and reportedly correlated with chronic airway hyperreactivity (31). As viral RNA can engage TLR7, RSV persistence may stimulate concurrent inflammation by TLR7, possibly driving a chronic inflammatory phenotype.

Here, we have interrogated the role of TLR7 in both acute and chronic RSV-induced pathogenesis with a focus on URT (nasal) and LRT (upper airways and lung) responses.

## 2 Results

### 2.1 TLR7 modestly regulates viral clearance in the URT to suppress localized inflammation

**Figure 1:**
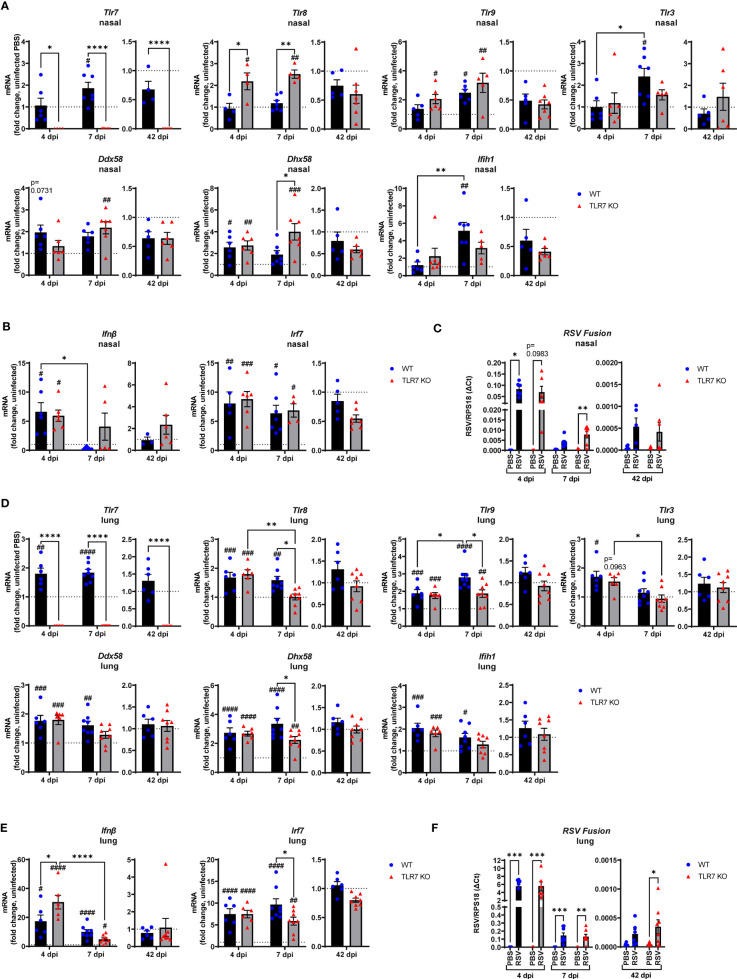
Gene expression analysis of pattern recognition receptors, type I IFN and viral titers in the nasal and lung tissue of RSV infected mice. WT C57BL/6 or TLR7 KO mice were infected with RSV (0.5-2x10^7^ PFUs) or PBS via intranasal administration. mRNA expression of **(A, D)** pattern recognition receptors, **(B, E)** type I IFN or **(C, F)** RSV Fusion gene was quantified in nasal or lung tissue after 4, 7 or 42 dpi. Responses are relative to RPS18 and expressed as a fold-change above uninfected controls of each mouse genotype. Data are expressed as mean ± SEM, n = 4-8 mice per experimental group from a single experiment. Statistical analysis was conducted using two-way ANOVA test followed by Tukey’s *post hoc* test for multiple comparison to compare differences between genotype or infection timepoints (*p < 0.05, **p < 0.01, ****p<0.0001). A Sidak’s *post hoc* test was performed to compare differences between uninfected controls (fold change of 1) and respective infected groups for each genotype (***p<0.001, ^#^p < 0.05, ^##^p < 0.01, ^###^p < 0.001, ^####^p < 0.0001).

To assess the impact of TLR7 during acute and chronic RSV infection, WT and TLR7 KO mice were infected with RSV and airway and lung inflammation subsequently measured at early (4 or 7 dpi) or late (42 dpi) timepoints to model different phases of the disease. We first investigated nasal tissue to understand the contribution of TLR7 to antiviral responses in the URT. Gene expression analysis of the nasal tissue revealed that early infection (4 dpi) did not alter TLR expression, including that of TLR7 despite virus being present and evoking elevated IRF-7 and IFN-β expression (**Figures 1A, B**). Indeed, the expression of RIG-I/DDX58 and its positive regulator LGP2/DHX58 were higher at this timepoint. Deletion of TLR7 did not change the levels of virus, IFN-β or DHX58, although the expression of TLR8 and TLR9 was significantly higher than in WT mice, suggesting the presence of active TLR7 may suppress other TLR activation/function. TLR7 expression rose significantly at 7 dpi in WT mice, although this was not associated with any changes in type I IFN (**Figure 1A**). Interestingly, significant levels of virus were detected in the nasal tissue of TLR7 KO mice at 7 dpi (**Figure 1C**) and this coincided with significantly elevated expression of PRRs in these mice. Furthermore, we were able to detect viral RNA in the nasal tissue of some mice of either genotype at 42 dpi (**Figure 1C**, **Supplementary Table S1**), although PRR expression had returned to near uninfected levels by this time.

The expression of TLR7 in the lungs of WT mice increased significantly after RSV infection, at 4 and 7 dpi, but did not appear to persist chronically, despite the detection of residual viral mRNA at 42 dpi (**Figure 1D**). Upregulation of various TLRs and RLRs similarly occurred in the presence or absence of TLR7 at 4 dpi and were retained at 7 dpi, but only in WT mice (**Figure 1D**). PRR regulation was significantly suppressed in the lungs of TLR7 KO mice at 7 dpi. Unlike in the nasal tissue, this difference did not impact acute clearance of RSV in the lungs as viral titres remained the same in both genotypes (**Figure 1F**), implying that antiviral responses were not compromised in the LRT of TLR7 KO mice. Indeed, IFN-β and IRF-7 expression in the lung were elevated upon RSV infection at 4 dpi in both genotypes, with significantly higher IFN-β levels in TLR7 KO mice (**Figure 1E**). Resolution of type I IFN expression was evident at day 7 and occurred more dramatically in TLR7 KO mice. The low level mRNA viral transcripts detected at 42 dpi were consistent with previous observations of chronic RSV persistence in the lungs (32) and a statistically significant higher viral titre in the lung was observed in TLR7 KO mice at 42 dpi (**Figure 1F**, **Supplementary T** **able S1**).

**Figure 2:**
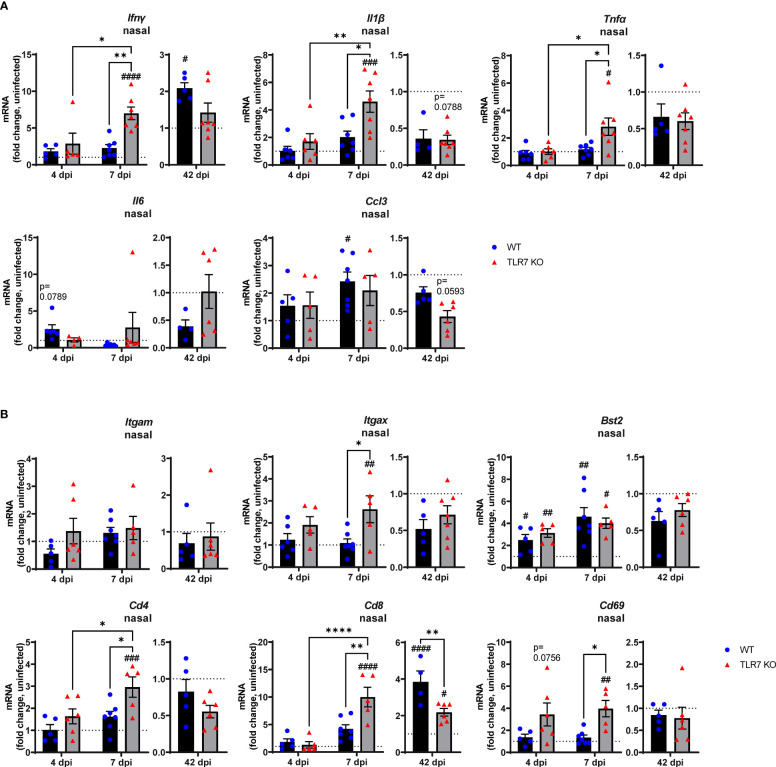
TLR7 suppresses acute inflammatory signaling in the nasal tissue following RSV infection. WT C57BL/6 or TLR7 KO mice were infected with RSV (0.5-2x10^7^ PFUs) or PBS via intranasal administration. Nasal tissue mRNA expression of various **(A)** proinflammatory cytokines and **(B)** immune specific markers was quantified after 4, 7 or 42 dpi. Responses are relative to RPS18 and expressed as a fold-change above uninfected controls of each mouse genotype. Data are expressed as mean ± SEM, n = 4-8 mice per experimental group from a single experiment. Statistical analysis was conducted using two-way ANOVA test followed by Tukey’s *post hoc* test for multiple comparison to compare differences between genotype or infection timepoints (*p < 0.05, **p < 0.01, ****p<0.0001). A Sidak’s *post hoc* test was performed to compare differences between uninfected controls (fold change of 1) and respective infected groups for each genotype (^#^p < 0.05, ^##^p < 0.01, ^###^p < 0.001, ^####^p < 0.0001).

These data indicate that RSV infection engages various IFN-stimulating sensors in the URT where TLR7 modestly contributes to viral clearance. Interestingly, we failed to detect upregulation of inflammatory response genes in the nasal tissue of WT mice in response to acute RSV infection. In contrast, a significant elevation of various Th1 inflammatory markers IFNγ, IL-1β and TNFα was evident in the nasal tissue of TLR7 KO mice at 7 dpi (**Figure 2A**). This correlated with the increased expression of the DC marker CD11c/ITGAX as well as the T cell markers CD4, CD8 and CD69 (**Figure 2B**). Expression of the monocyte/macrophage/granulocyte marker CD11b/ITGAM was unaltered in both mouse genotypes and was accompanied by insignificant increases in CCL3. Additionally, upregulation of PDCA-1/BST2 expression (commonly expressed by pDCs and B cells) was consistent in both mouse genotypes during acute infection (**Figure 2B**). Strikingly, both WT and TLR7 KO mice displayed elevated nasal expression of CD8 at 42 dpi but this was significantly dampened in TLR7 KO mice (**Figure 2B**). Furthermore, increased IFNγ expression was additionally detected in the nasal tissue of WT mice at 42 dpi but not in TLR7 KO mice.

These analyses imply that RSV infection induces a dominant antiviral response in the URT of WT mice, and suggests a potential suppression mechanism of acute Th1 cytokine responses by TLR7 in the URT (up to 7 dpi), which likely contributes to the observed chronic persistence of a functional CD8+ cytotoxic T cell and IFNγ response in the URT at day 42.

### 2.2 TLR7 potentiates enhanced acute and chronic airway inflammation following RSV infection

**Figure 3:**
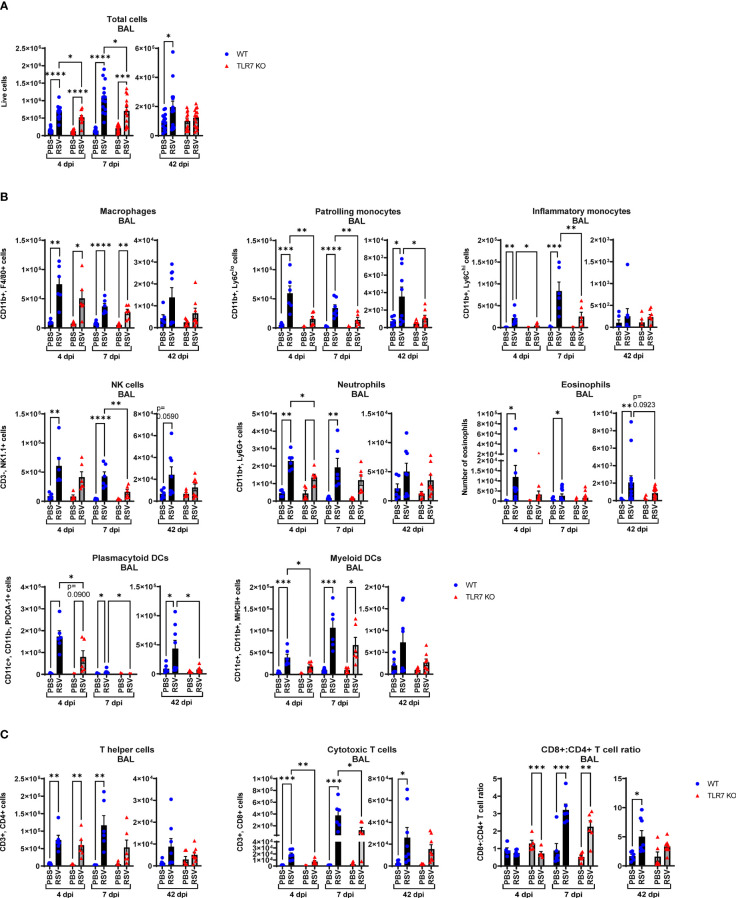
RSV-induced airway immune infiltration is reduced in TLR7 KO mice. WT C57BL/6 or TLR7 KO mice were infected with RSV (0.5-2x10^7^ PFUs) or PBS via intranasal administration and analysis performed after 4, 7 or 42 dpi. **(A)** Airway inflammation was assessed by counting the total number of live cells isolated from the bronchoalveolar lavage (BAL) fluid. Data are expressed as mean ± SEM, n = 12-16 mice per experimental group from three independent experiments. Immune cell populations in the airways collected in the BAL fluid were determined by flow cytometry. **(B)** Innate immune cell types: interstitial macrophages (CD11b^+^ F4/80^+^), patrolling monocytes (CD11b^+^ Ly6C^lo^), inflammatory monocytes (CD11b^+^ Ly6C^hi^), NK cells (CD3^-^ NK1.1^+^), neutrophils (CD11b^+^ Ly6G^+^), plasmacytoid DCs (CD11c^+^ CD11b^-^ PDCA-1^+^) and myeloid DCs (CD11c^+^ CD11b^+^ MHCII^+^). Numbers of differentially stained eosinophils was determined by counting 500 cells from random fields by standard morphological criteria relative to the total number of isolated cells. **(C)** Adaptive T cell subtypes: T helper (CD3^+^ CD4^+^) and cytotoxic T cells (CD3^+^ CD8^+^). Cell populations were measured as absolute number of CD45^+^ population per total live cells. Data are expressed as mean ± SEM, n = 5-8 mice per experimental group from two independent experiments. Statistical analysis was conducted using two-way ANOVA test followed by Tukey’s *post hoc* test for multiple comparison test (*p < 0.05, **p < 0.01, ***p<0.001, ****p<0.0001).

Our data thus far indicated that TLR7 assists with viral clearance in the URT but its antiviral effects in the LRT are dispensable. Rather, better resolution of type I IFN in the LRT was achieved in the absence of TLR7 without compromising viral clearance. TLR7 also initiates important proinflammatory signals, which we found were suppressed in the URT, and given the apparent TLR7-dependent antiviral persistence in the LRT, sustained TLR7 activation (as observed by elevated lung TLR7 expression at 7 dpi) may contribute to hyperinflammatory responses that can potentially exacerbate LRT pathology. Infiltration of immune cells as a measure of inflammation into the large airways was assessed by bronchoalveolar lavage (BAL) across acute and chronic infection. Airway inflammation peaked at 7 dpi and was significantly lower in TLR7 KO mice across acute timepoints (**Figure 3A**). This result was in concordance with type I IFN suppression in the nasal tissue at 7 dpi and is consistent with a sequence of antiviral resolution in the URT preceding the dissemination of a robust inflammatory response in the LRT. Interestingly, a significant population of immune cells were retained in the airways of infected WT mice at 42 dpi, but not in TLR7 KO mice. Considering the equivalent level of virus detected acutely in the lungs, this observation indicates that TLR7 enhances airway inflammation without improving viral clearance.

Flow cytometric labelling of immune cell markers revealed that RSV infection induced an early airway influx of innate immune responder cells, comprised of macrophages, monocytes, neutrophils, natural killer (NK) and DCs at 4 dpi (**Figure 3B**). Eosinophilia also accompanied infection. There were significantly less pDCs and myeloid dendritic cells (mDCs), macrophages and monocytes in RSV-infected TLR7 KO mice at this timepoint. The frequency of patrolling and inflammatory monocytes in the airways was also significantly reduced in infected TLR7 KO mice (**Supplementary Figure S1A**). By 7 dpi, the numbers of Ly6C^hi^ inflammatory monocytes and mDCs increased in WT mice while other innate immune cells had decreased. Similar to the inflammation at 4 dpi, the airways of TLR7 KO mice contained less innate immune cell numbers at 7 dpi with significantly lower NK cells and monocytes. There was no difference in the composition of cells in the airways of infected mice between genotypes at 7 dpi (**Supplementary Figure S1A**). Interestingly, a significant number of pDCs, Ly6C^lo^ patrolling monocytes and eosinophils persisted in the airways of infected WT mice at 42 dpi, with non-significant trends of macrophage and NK cell populations also present (**Figure 3B**). These lingering cells were absent in infected TLR7 KO mice at 42 dpi.

Analysis of the adaptive T cell response revealed the migration of a dominant population of CD4+ helper T cells to the airways of infected mice of both genotypes at 4 dpi, but the T cell response eventually shifted towards a CD8+ cytotoxic phenotype by 7 dpi (**Figure 3C**, **Supplementary Figure S1B**). Despite RSV infection increasing CD8+ cytotoxic T cells to similar frequencies in both genotypes, approximately half as many CD8+ cytotoxic T cells were detected in TLR7 KO mice at 7 dpi, although this did not affect overall viral clearance. Interestingly, CD8+ cytotoxic T cells persisted in the airways of infected mice at 42 dpi at similar frequencies in both genotypes, but total cell numbers were lower in TLR7 KO mice. Furthermore, we noted that WT mice exhibited a greater CD8+:CD4+ T cell ratio at 42 dpi compared to TLR7 KO mice. These data reveal an acute TLR7-dependent enhanced total cellular immune response in the airways of RSV-infected mice and a chronic persistence of a subset of immune cells namely CD8+ cytotoxic T cells, NK cells, patrolling monocytes, eosinophils and pDCs.

### 2.3 RSV infection provokes TLR7-dependent lung pathology

**Figure 4:**
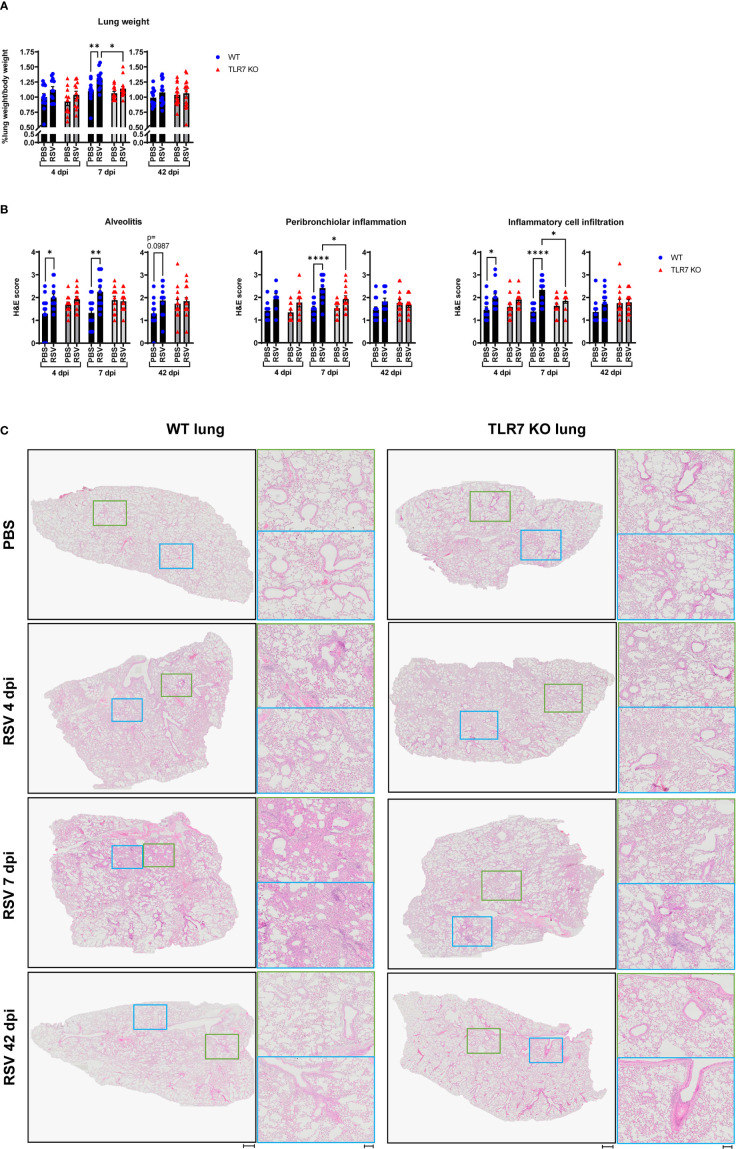
TLR7 KO mice have reduced lung pathology following RSV infection. WT C57BL/6 or TLR7 KO mice were infected with RSV (0.5-2x10^7^ PFUs) or PBS via intranasal administration, then lung pathology was assessed after 4, 7 or 42 dpi. **(A)** Pulmonary oedema was assessed by measuring the wet lung weight to bodyweight ratio. **(B)** Lung histopathological analysis was assessed for alveolitis (alveolar inflammation of parenchyma), total inflammatory cell infiltrate and peribronchiolar inflammation (inflammation around bronchiolar airway wall) using a scoring system of 0–5 for each individual mouse, where higher numbers indicate increased severity. Each section was scored blindly by two independent assessors. **(C)** Representative images displaying lung inflammation from paraffin embedded lung sectioned (4 μm) longitudinally and stained with H&E. Scale bars represent 1 mm in the overview and 100 μm for the zoomed images. Data are expressed as mean ± SEM, n = 12-16 mice per experimental group from three independent experiments. Statistical analysis was conducted using two-way ANOVA test followed by Tukey’s *post hoc* test for multiple comparison test (*p < 0.05, **p < 0.01, ****p<0.0001).

Our analysis of persistent immune cell infiltration detected in the BAL fluid indicated that this model of RSV infection induces chronic inflammation of the larger airways in the LRT that is driven by TLR7. As mentioned earlier, severe RSV infection of the LRT can manifest in some individuals leading to bronchiolitis and pneumonia. RSV infection induced lung oedema in WT mice at 7 dpi, as evidenced by significantly heavier lung weights compared to uninfected lungs, but this oedema was absent in TLR7 KO mice infected with RSV (**Figure 4A**). This may be an indication of the greater infiltration of inflammatory cells to the lungs of TLR7 expressing mice following infection, consistent with enhanced airway infiltration.

Haematoxylin and eosin (H&E) staining of lung sections was conducted to assess the level of inflammation within the lung tissue. Acute alveolitis was evident in RSV-infected WT mice while TLR7 KO mice showed no difference in alveolar inflammation with infection (**Figures 4B, C**). Inflammation into the alveolar wall near the bronchioles peaked at 7 dpi in WT mice whereas in TLR7 KO mice there was significantly less infiltration of inflammatory cells around the bronchioles. Additionally, RSV infection resulted in an insignificant trend towards chronic alveolitis at 42 dpi in WT mice but this was not evident in TLR7 KO mice. There were overall fewer inflammatory cells infiltrating the lung tissue of TLR7 KO mice compared to WT mice during acute infection. No significant differences in the degree of immune infiltration of infected lungs were detected at 42 dpi in either mouse genotype, suggesting a resolution of immune lung infiltration, at least by H&E observation. This histological analysis indicates that RSV infection induces an infiltration of cells into the deep airway spaces of the lung, which is likely to be TLR7-dependent, leading to chronic alveolitis and enhanced bronchiolar inflammation.

**Figure 5:**
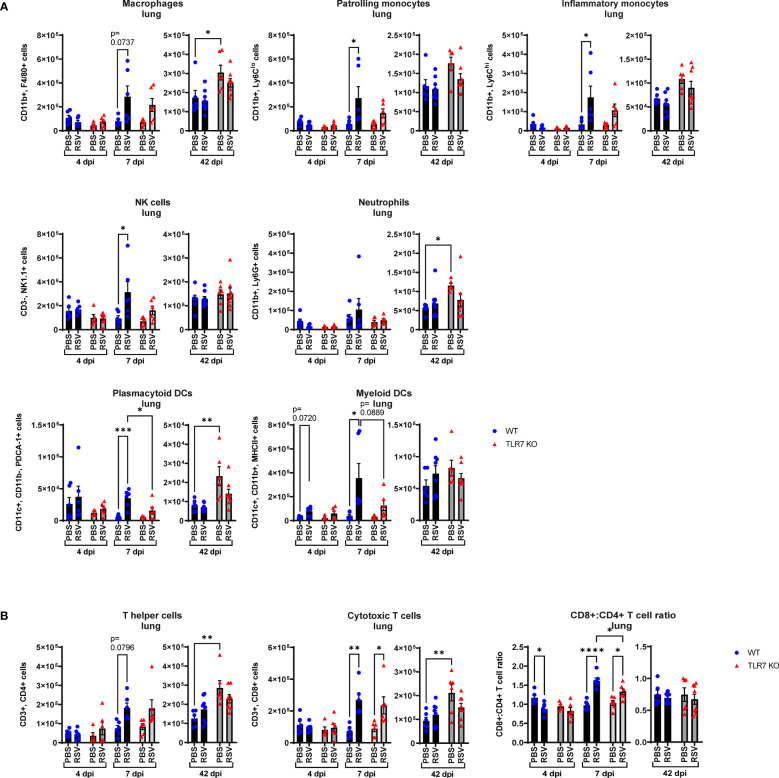
RSV-induced lung infiltration of innate immune cells is reduced in TLR7 KO mice. WT C57BL/6 or TLR7 KO mice were infected with RSV (0.5-2x10^7^ PFUs) or PBS via intranasal administration. Immune cell populations in lung tissue were determined by flow cytometry after 4, 7 or 42 dpi. **(A)** Innate immune cell types: interstitial macrophages (CD11b^+^ F4/80^+^), patrolling monocytes (CD11b^+^ Ly6C^lo^), inflammatory monocytes (CD11b^+^ Ly6C^hi^), NK cells (CD3^-^ NK1.1^+^), neutrophils (CD11b^+^ Ly6G^+^), plasmacytoid DCs (CD11c^+^ CD11b^-^ PDCA-1^+^) and myeloid DCs (CD11c^+^ CD11b^+^ MCHCII^+^). **(B)** Adaptive T cell types: T helper (CD3^+^ CD4^+^) and cytotoxic T cells (CD3^+^ CD8^+^). Cell populations are measured as absolute number of CD45^+^ population per 10,000 counting beads. Data are expressed as mean ± SEM, n = 5-8 mice per experimental group from two independent experiments. Statistical analysis was conducted using two-way ANOVA test followed by Tukey’s *post hoc* test for multiple comparison test (*p < 0.05, **p < 0.01, ***p<0.001, ****p<0.0001).

We further analyzed the infiltrating immune cell types in the lungs following RSV infection at both acute and chronic stages. Significant changes in immune cell numbers (monocytes, DCs and NK cells) in the lungs of WT mice following infection were evident at 7 dpi (**Figure 5A**). The lungs of infected TLR7 KO mice consisted of significantly less pDCs, and a trend towards less NK cells, mDCs monocytes at 7 dpi (**Figure 5A**). Populations of CD4+ and CD8+ T cell subtypes were similar in the infected lungs of either mouse genotype. While infection at day 7 increased the CD8+:CD4+ T cell ratio in the lungs of both genotypes, this was significantly lower in TLR7 KO mice (**Figure 5B**). This, in conjunction with the immune analysis of the airways, implied an overall reduced T cell effector response in the absence of TLR7, without compromising viral clearance. However, unlike in the upper airways of the LRT, infection did not result in any chronic increases of immune cells in the lungs at 42 dpi.

These data show that RSV infection causes a TLR7-dependent infiltration of inflammatory cells to the deeper lung tissue and that the chronic inflammatory phenotype, in addition to the acute phase of infection, is mainly localized to the upper airways of the LRT.

### 2.4 TLR7 drives an exacerbated Th1/Th2 proinflammatory profile in the lungs following RSV infection

**Figure 6:**
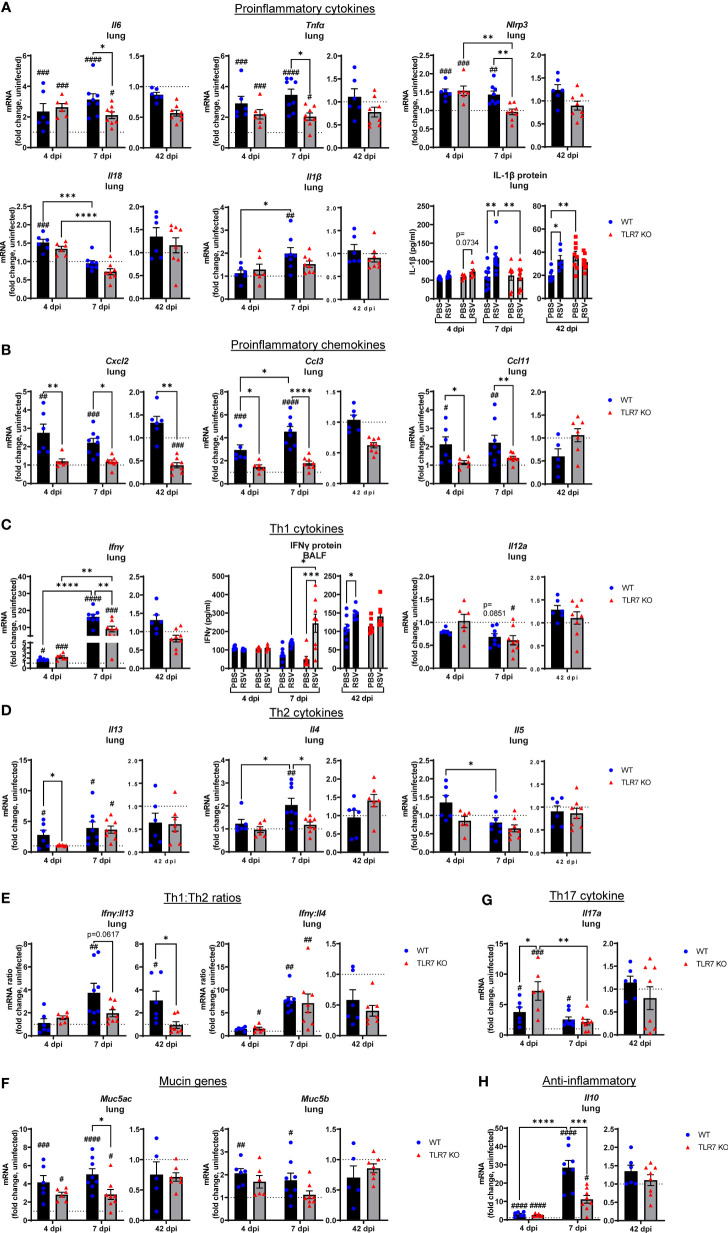
Reduced pro-inflammatory mediators in the lungs of TLR7 KO mice. Lung mRNA expression from WT C57BL/6 or TLR7 KO mice infected with RSV (0.5-2x10^7^ PFUs) or PBS was analyzed after 4, 7 or 42 dpi. **(A)** Proinflammatory cytokines, **(B)** proinflammatory chemokines, **(C)** Th1 cytokines, **(D)** Th2 cytokines, **(E)** Th1:Th2 cytokine ratios, **(F)** mucin genes, **(G)** Th17 and **(H)** anti-inflammatory cytokines were measured. Proteins levels of **(A)** IL-1β from the lung or **(C)** IFNγ from the BALF were also quantitated by ELISA. Responses are relative to RPS18 and expressed as a fold-change above uninfected controls of each mouse genotype. Data are expressed as mean ± SEM, n = 5-8 mice per experimental group from a single experiment. Statistical analysis was conducted using two-way ANOVA test followed by Tukey’s *post hoc* test for multiple comparison to compare differences between genotype or infection timepoints (*p < 0.05, **p < 0.01, ****p<0.0001). A Sidak’s *post hoc* test was performed to compare differences between uninfected controls (fold change of 1) and respective infected groups for each genotype (***p<0.001, ^#^p < 0.05, ^##^p < 0.01, ^###^p < 0.001, ^####^p < 0.0001).

To further dissect the immunopathological mechanism driven by TLR7-mediated hyperinflammatory responses in the LRT during RSV infection, we analyzed the expression of key Th1 and Th2 cytokine mediators as alterations in these responses can influence the severity of RSV infection and risk of subsequent respiratory illness, particularly in early life (33, 34). Early inflammasome activation was induced 4 dpi, as evidenced by increased expression of NLRP3 and IL-18, as well as IL-6 and TNFα upregulation in the lungs of both mouse genotypes (**Figure 6A**). Lung IL-1β gene and protein expression were higher at 7 dpi in WT mice, as were NLRP3, IL-6 and TNFα gene levels, however these proinflammatory markers were suppressed in the absence of TLR7. This also correlated with a TLR7-dependent alteration in chemotactic factors: where infection raised the levels of CXCL2, CCL3 and CCL11 chemokines at 4 and 7 dpi in WT mice, but these responses were blunted in TLR7 KO mice (**Figure 6B**), most likely accounting for the reduced airway infiltration of neutrophils, macrophages and eosinophils in TLR7 KO mice.

Inflammasome activation has direct effects on shaping the Th1, Th2 and Th17 immune responses (35). Infection promoted early expression of the Th1 cytokine IFNγ in the lungs at day 4 that dramatically rose by more than 10-fold at 7 dpi (**Figure 6C**). Gene expression of another Th1 cytokine IL-12 in the lung did not change with infection. The levels of IFNγ were similar between genotypes at 4 dpi, but TLR7 KO mice expressed significantly less IFNγ at 7 dpi. This observation in the lungs of TLR7 KO mice at 7 dpi contrasts with the nasal tissue analysis in which these mice displayed higher IFNγ expression at the same timepoint (**Figure 2A**). However, secreted protein levels of IFNγ in the BALF at 7 dpi (**Figure 6C**) mirrored the nasal tissue data demonstrating higher IFNγ levels in the airways of TLR7 KO mice, consistent with delayed viral clearance in the URT. This suggests that the loss of TLR7 enhances Th1 responses in the URT, but limits hyperactivation in the LRT. In regard to Th2 cytokines, higher IL-13 expression was detected at 4 dpi in WT lungs and was retained at 7 dpi (**Figure 6D**). This early IL-13 response at 4 dpi was blunted in TLR7 KO mice, but rose to similar levels to WT mice at 7 dpi. In contrast, the IL-4 response was not evident until 7 dpi and was only detected in the WT lung.

The expression profile of the panel of inflammatory cytokines and chemokines analyzed was unaltered in the infected lung tissue of both genotypes of mice at 42 dpi. This is in contrast to the URT, where only infected WT mice showed significantly higher IFNγ expression in the nasal tissue (**Figure 2A**) or BALF (**Figure 6C**) at the day 42 timepoint. Analysis of the IFNγ:IL-13 ratio as an indicator of Th1 versus Th2 response supported the notion of a dominant Th1 response in the lungs of infected WT mice at day 7, which was retained at day 42 (**Figure 6E**). This analysis also suggested that the lungs of infected TLR7 KO mice did not exhibit a dominant Th1 or Th2 phenotype at either acute or chronic timepoints. Conversely, the IFNγ:IL-4 ratio, as an alternative measure of Th1 versus Th2, was higher in infected TLR7 KO mice at 4 dpi, although eventually rose to the same degree in both genotypes at 7 dpi, supporting an overall Th1 dominant response at early timepoints. Despite a significant influx of Th2-dominant eosinophils to the airways of WT mice (**Figure 3B**), IL-5 expression did not change during infection (**Figure 6D**). Furthermore, acute RSV infection increased expression of mucin genes Muc5b and Muc5ac in the lung, suggesting enhanced mucus production and goblet cell hyperplasia with infection (**Figure 6F**). Consistent with the suppressed inflammatory profile, Muc5ac expression was significantly reduced in TLR7 KO mice at 7 dpi.

IL-17A levels were also high at 4 dpi although expression was significantly higher in TLR7 KO mice (**Figure 6G**), suggesting these mice had an enhanced Th17 profile during early infection, but this response was resolved in both genotypes by day 7. The lung profile of IL-10, which is considered to exert suppressive effects on inflammation, mirrored the IFNγ response. Infection induced equivalent levels of IL-10 at 4 dpi in both genotypes, which then rose dramatically in WT mice at 7 dpi, but this increase was significantly less in TLR7 KO mice (**Figure 6H**). This suggests that the overall reduction of the Th1/Th2 response in the lungs of TLR7 KO mice is paralleled by the reduced need for inflammatory suppression signaling.

These data demonstrate that TLR7 promotes a profound hyperinflammatory response to RSV infection in the LRT following effective early antiviral responses by driving a strong Th1 dominant phenotype in the lung, which persists chronically. Loss of TLR7 improves the resolution of this inflammatory response (at 7 dpi) in the LRT by balancing the Th1/Th2 landscape that ultimately reduces the acute and chronic immunopathology caused by excess immune infiltration of the airways present in TLR7 proficient mice.

### 2.5 RSV infection induces a TLR7-dependent inflammatory response in alveolar macrophages

**Figure 7:**
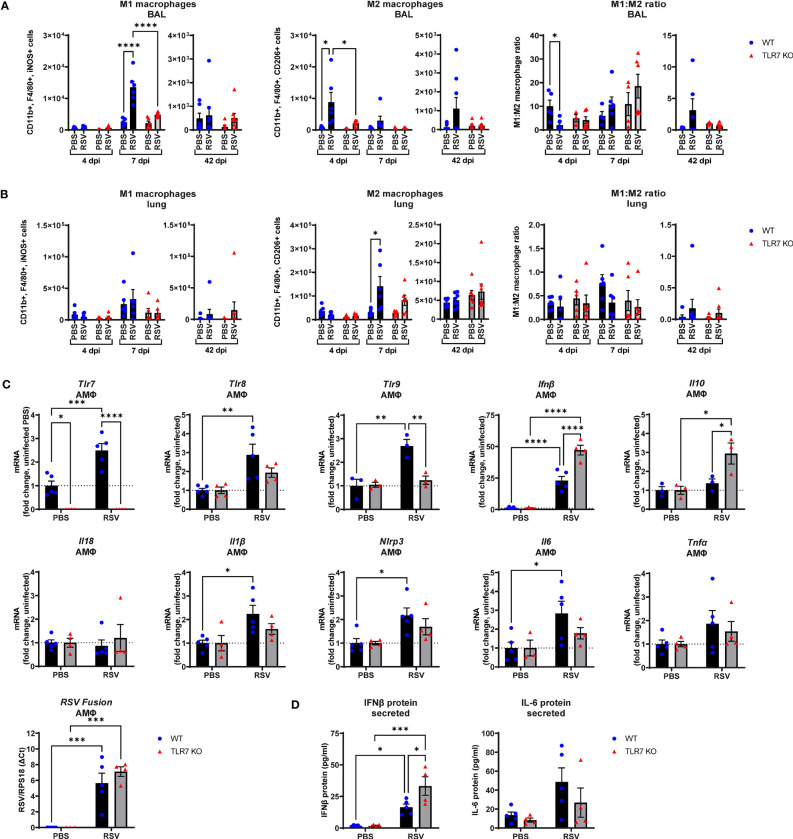
RSV infection stimulates proinflammatory responses in alveolar macrophages. WT C57BL/6 or TLR7 KO mice were infected with RSV (0.5-2x10^7^ PFUs) or PBS. After 4, 7 or 42 dpi, macrophage subtypes M1 “inflammatory” (CD11b^+^ F4/80^+^ iNOS^+^) and M2 “anti-inflammatory” (CD11b^+^ F4/80^+^ CD206^+^) were isolated from the **(A)** airways (BAL) or **(B)** lung tissue and assessed by flow cytometry. Cell populations were measured as absolute number of CD45^+^ population per 10,000 counting beads (lung) or total live cells (BAL). Data are expressed as mean ± SEM, n = 5-8 mice per experimental group from two independent experiments. **(C)** Alveolar macrophages (AMФ) were isolated and infected *ex vivo* with RSV (MOI 1) for 24 h. Expression of indicated genes was then performed, and is presented relative to RPS18 as a fold-change above uninfected controls of each genotype. **(D)** Secreted protein was also measured in the cell culture supernatant by ELISA. Data are expressed as mean ± SEM, n = 3-5 per treatment group from three independent experiments. Statistical analysis was conducted using two-way ANOVA test followed by Tukey’s *post hoc* test for multiple comparison test (*p < 0.05, **p < 0.01, ***p<0.001, ****p<0.0001).

The above experiments revealed that the RSV-induced inflammatory signal is predominantly derived from the LRT rather than the URT (nasal compartment), suggesting that immune infiltration to the upper airways is heavily influenced by the Th1/Th2 cytokine signals in the lung. As RSV disseminates to the LRT it commonly infects and stimulates pulmonary macrophages, although these cells do not efficiently support RSV replication but are important initiators of inflammation in the lung (36). Studies have also reported an indirect activation mechanism of alveolar macrophages (AMФ) by upstream pDCs via TLR7, which may drive a “cytokine storm” in the LRT (26, 27). Thus, Th1/Th2 signaling in the lung can likely influence the polarization of macrophage subtypes and vice versa. RSV infection induced an early Th2-skewed M2 “anti-inflammatory” macrophage response at 4 dpi in the airways that shifted to a Th1 dominant M1 “inflammatory” macrophage response at day 7 (**Figure 7A**). Both M1 and M2 macrophage responses were blunted in TLR7 KO mice across acute infection timepoints, consistent with the cytokine gene expression in the lung. Moreover, populations of M2 macrophages from lung tissue increased significantly in infected WT mice at 7 dpi while no significant increases in M2 populations were observed in TLR7 KO mice (**Figure 7B**). The M1 macrophage population and overall M1:M2 ratios did not change significantly in the lung following infection in either genotype. However, M1:M2 ratios in the upper airways suggested RSV infection induced an early Th2 dominant inflammatory response disseminating from the URT in a TLR7 dependent manner.

Additional experiments on naive AMФ isolated from WT or TLR7 KO mice and subsequently infected *ex vivo* with RSV were performed to assess the intrinsic effects of RSV on these cells. RSV induced a cell-intrinsic increase in TLR7, TLR8 and TLR9 expression in WT-derived AMФ, and this was accompanied by enhanced IFN-β expression, which was curiously higher in macrophages lacking TLR7, although the viral load remained equivalent (**Figure 7C**). Therefore, these results may account for the elevated IFN-β levels observed in the lungs of infected TLR7 KO mice at early timepoints (**Figure 1B**). Measurement of secreted IFN-β protein into the culture supernatant also revealed increased production of IFN-β protein by TLR7-deficient AMФ upon exposure to RSV compared to WT macrophages (**Figure 7D**). In addition, there was no significant increase in TLR8 or TLR9 expression observed in TLR7 KO macrophages. Infection boosted IL-1β, IL6 and NLRP3 levels in TLR7 expressing AMФ but these proinflammatory markers were suppressed upon TLR7 deficiency. Additionally, anti-inflammatory IL-10 was only significantly elevated in TLR7-deficient AMФs. We were unable to detect either basal or stimulated gene expression of IL-4, IL-5, IL-13 and IFNγ in AMФ. These experiments reveal that direct RSV infection of AMФ preferentially activates a TLR7-dependent proinflammatory response that is ineffectively regulated and somewhat suppresses type I IFN.

### 2.6 RSV infection provokes TLR7-dependent chronic airway hyperreactivity that correlates with CD8+:CD4+ T cell ratios in the airways

**Figure 8:**
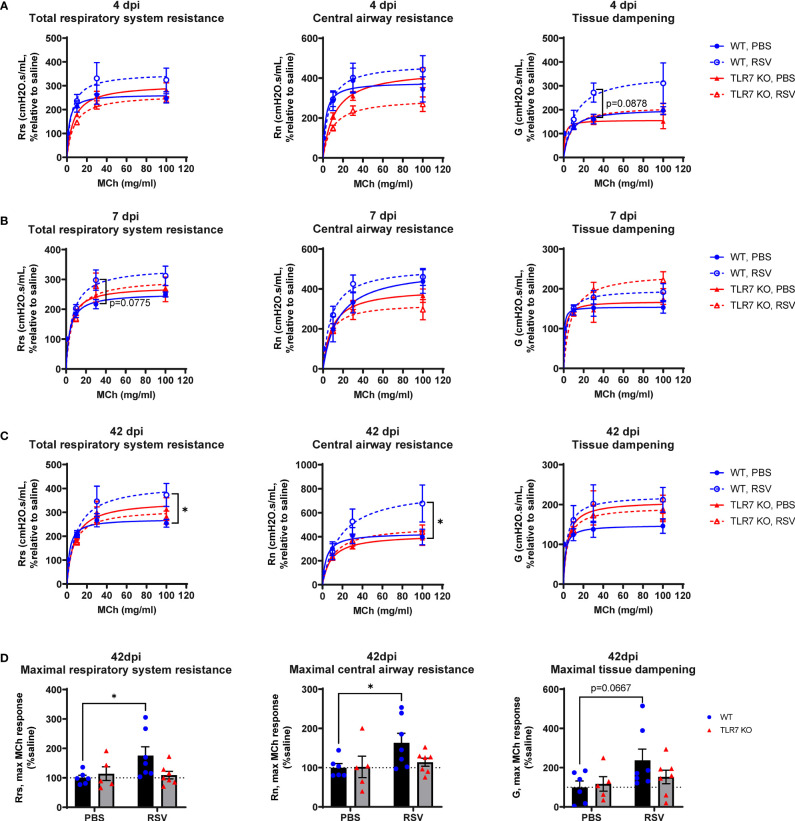
TLR7 KO mice are spared from RSV-induced chronic airway hyperreactivity. WT C57BL/6 or TLR7 KO mice were infected with RSV (0.5-2x10^7^ PFUs) or PBS. Mice were anaesthetized after 4, 7 or 42 dpi, connected to the Flexivent FX1 system and mechanically ventilated at 300 breaths per minute. Lung function was performed in response to increasing doses of nebulized methacholine (MCh; 0, 3, 10, 30 and 100 mg/ml). Dose response curves of central airways resistance (Rn, Newtonian resistance), total respiratory system resistance (Rrs) and tissue dampening (G) is presented relative to 0 mg/ml MCh (saline) after **(A)** 4, **(B)** 7 or **(C)** 42 dpi. **(D)** Maximal resistance (100 mg/ml MCh) parameters at 42 dpi relative to 0 mg/ml MCh (saline) are shown. Data are expressed as mean ± SEM, n = 5-8 mice per experimental group from two independent experiments. Statistical analysis was conducted using two-way ANOVA test followed by Tukey’s *post hoc* test for multiple comparison test (*p < 0.05).

**Figure 9:**
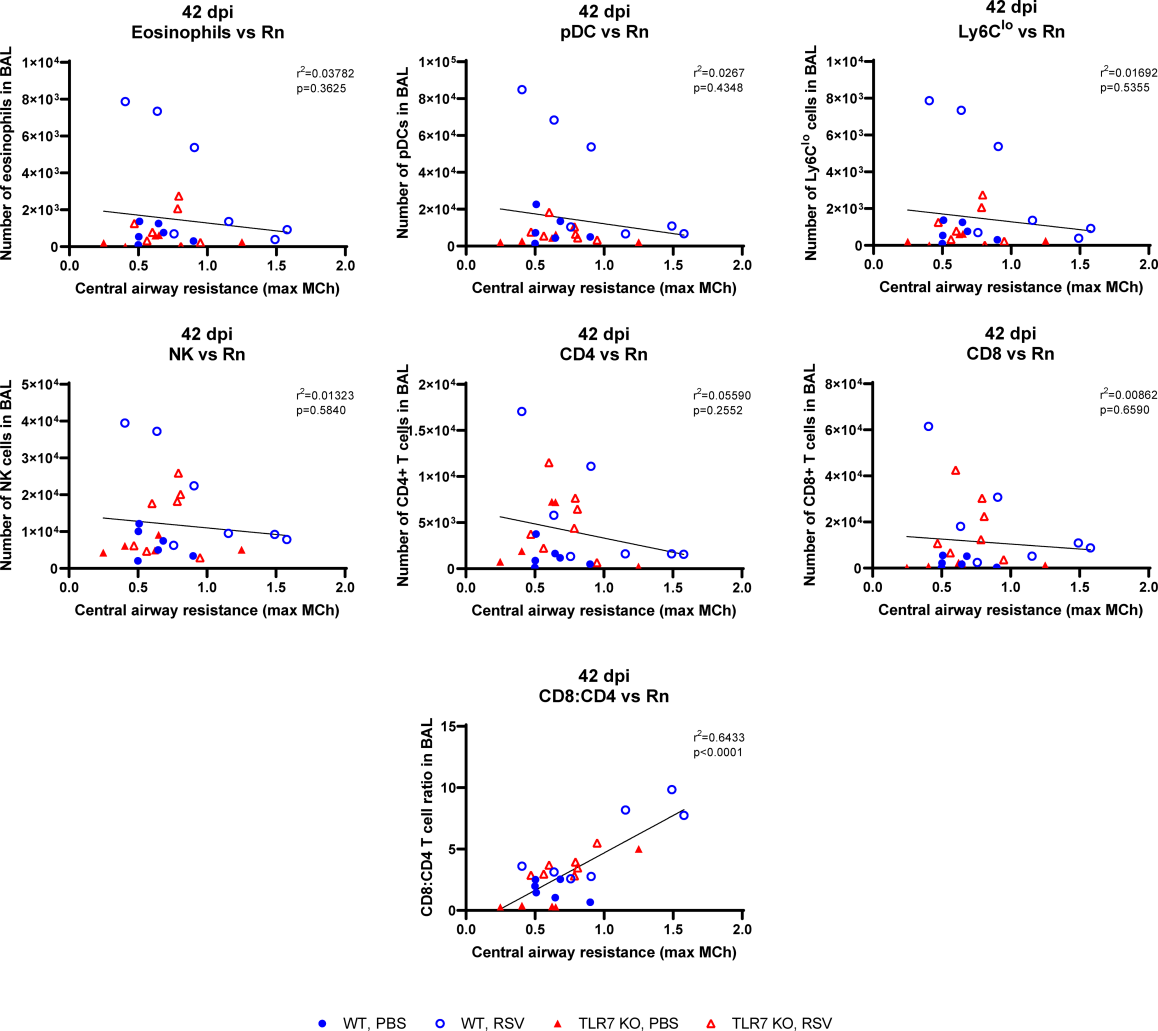
CD8+:CD4+ T cell ratios in the airways correlate with airway hyperresponsiveness. Correlations at 42 dpi were made of the maximal central airways resistance (100 mg/ml MCh) with the number of immune cells present in the airways (BAL) of each mouse. Data are expressed as mean ± SEM, n = 5-8 mice per experimental group from two independent experiments. Statistical analysis was conducted using a simple linear regression test.

We next determined if the chronic persistence of airway inflammation offered by TLR7 contributed to airway hyperreactivity as a functional consequence of poorly resolved acute hyperinflammatory responses in the LRT. Mice were subjected to methacholine (MCh) challenge after 4, 7 or 42 dpi then Newtonian resistance (Rn, central airways resistance), tissue dampening (G, small airway and alveolar resistance) and total lung resistance (Rrs, total airway resistance) were measured. WT mice infected with RSV displayed overall higher resistance parameters than uninfected mice at 4 and 7 dpi (**Figures 8A, B**). Importantly, central airway and total lung resistance were significantly higher in infected WT mice at 42 dpi, illustrating the presence of chronic airway and lung hyperreactivity (**Figures 8C, D**). There was also a non-significant trend for enhanced alveolar (G) resistance in infected WT mice at 42 dpi. In contrast, uninfected and RSV-infected TLR7 KO mice displayed similar responses to MCh for all resistance parameters at 42 dpi, indicating these mice did not experience airway hyperreactivity. We then stratified the number of immune cells retained in the airways at 42 dpi with the extent of central airway hyperreactivity at maximal MCh (100 mg/mL). A positive correlation was identified between the CD8+:CD4+ T cell ratios in the airways and maximum airway hyperreactivity (**Figure 9**).

These experiments demonstrate that TLR7 drives chronic airway hyperreactivity following RSV infection and this correlates with the CD8+:CD4+ T cell ratio in the airways.

## 3 Discussion

Inflammation is critical for regulating viral clearance and establishing immunological memory, however uncontrolled, widespread inflammation may prolong disease pathology resulting in tissue damage, disease progression, and even autoimmunity. TLR7 is an important immune sensor and key driver of inflammation following viral infection that has been implicated in hyperinflammatory responses in autoimmunity, influenza and SARS-CoV2 infection (26, 27, 37, 38). We reasoned that TLR7 plays a significant role in controlling the URT infection and the immunopathology provoked by RSV in the LRT, where chronic respiratory disease manifests.

**Figure 10:**
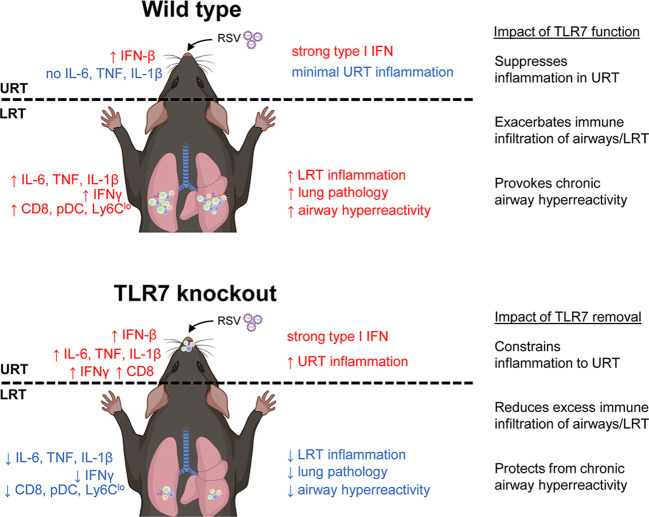
Proposed mechanism of TLR7-dependent LRT disease caused by RSV infection. In wild type (TLR7-proficient mice), RSV infection induces a strong type I IFN response in the URT (most likely modulated by RLRs) with minimal inflammation. However, inflammation of the LRT ensues involving infiltration of various innate and T cell immune subtypes that is potentiated by TLR7 and resolution of this is lacking resulting in persistent LRT inflammation, lung pathology and chronic airway hyperreactivity. In contrast, infection of TLR7 knockout mice induces type I IFN and persistent inflammatory signaling in the URT, implying that TLR7 has anti-inflammatory properties in the nasal tissue. The resulting inflammation of the LRT is thereby subdued and this reduces lung pathology and spares mice from chronic airway hyperreactivity.

Mice infected with RSV developed airway hyperreactivity several weeks after infection, consistent with previous neonatal and adult mouse models of RSV (39, 40). Strikingly, the absence of TLR7 prevented RSV-infected mice from developing chronic airway hyperreactivity. Our study showed that the acute phase of RSV infection established a TLR7-dependent inflammatory niche within the LRT that facilitated the development of chronic respiratory disease. While TLR7 suppressed inflammatory markers in the nasal tissue, this enhanced the progression of inflammation and immune infiltration in the LRT, which added little benefit to the host response to infection, but rather expedited long-term immunopathological consequences. In contrast, the absence of TLR7 in the URT provoked a localized inflammatory response in the nasal tissue that modestly impacted viral clearance, but prevented the dissemination and establishment of a hyperinflammatory environment in the LRT. Overall, these observations illustrated a sub-optimal resolution of the host immune response in the LRT that is driven by TLR7, which contributes to hyperinflammatory signaling, lung pathology and persistent immune infiltration of the airways, leading to chronic airway hyperreactivity (**Figure 10**). It is unclear whether this effect can occur upon direct engagement of TLR7 in the absence of a productive infection, although we reason that this sustained immune response, rather than persistent viral mRNA, induced chronic airway hyperreactivity as mice lacking TLR7 effectively resolved inflammation of the LRT and did not experience airway hyperreactivity.

TLR7 paradoxically contributed different immune responses in the URT and LRT in response to RSV infection. We did not detect a strong proinflammatory signal in the nasal tissue during acute infection implying that the immune response in the URT was predominantly antiviral, which was likely propagated early by cytosolic RLRs. Loss of TLR7 modestly impeded clearance of the virus in the nasal tissue as viral titres were higher in TLR7 KO mice at 7 dpi, and this coincided with the upregulation of IFNγ, IL-1β and TNFα, as well as CD8+ T cells. In contrast, early (day 4) antiviral responses in the LRT of TLR7 KO mice were not impaired. This was accompanied by intact levels of proinflammatory mediators and concomitant airway infiltration of NK cells, macrophages and CD4+ T cells, with no observed impact on viral titres in the lung. This is consistent with the engagement of various PRRs driving the antiviral responses to RSV infection (41–43) and illustrates that, unlike in the URT, TLR7 is dispensable for antiviral effects in the LRT in this model. Only TLR7 expressing mice displayed heightened inflammatory responses, lung pathology and immune infiltration of the airways in the LRT at 7 dpi; suggesting that the initial PRR response to infection combined with the secondary TLR7 response to effect a dual immune activation scenario, which might explain the heightened inflammation. Moreover, sustained levels of some inflammatory markers implicated in driving immunopathology, for instance elevated NLRP3 inflammasome activity, or NK cell or M1 macrophage populations (44–46), were lessened in the LRT of TLR7 KO mice. This effect was probably facilitated by the lack of CXCL2 and CCL3 upregulation in these mice although the observed lack of effect on viral titres highlights LRT viral clearance was not compromised.

Our data also imply that the RSV-induced inflammatory response is compartmentalized to the URT in TLR7 KO mice, thereby sparing the LRT from exacerbated immune infiltration and the development of critical lung pathology. Markers of inflammation were significantly elevated in the nasal tissue of TLR7 KO mice at 7 dpi but there was reduced immune infiltration to the rest of the airways, suggesting that the inflammatory response was localized to the URT. Importantly, less pDCs were present in the upper airways of the LRT early during infection, which likely influenced the airway responses of AMΦ and CD8+ T cells, as has been reported for SARS-CoV2 (26) and RSV (47) respectively. As such, we found the TLR7-mediated response to RSV in AMΦ primarily promoted inflammatory signaling. Thus, reduced AMΦ activation in TLR7 KO mice (a result of lower pDC stimulation or viral dissemination), is likely to limit inflammation in the LRT. CCL3-mediated chemotaxis has also been noted to influence the migration and function of CD8+ T cells to sites of infection following RSV exposure (48). Nasal aspirates obtained from infants hospitalized with severe RSV-induced bronchiolitis contained higher levels of CCL3, neutrophils, monocytes and Th2-CD8+ T cell subtypes, suggesting that these markers in the nasal tissue correlate with the severity of pathology in the LRT (49, 50). Interestingly, the expression of CCL3 in the nasal tissue of TLR7 KO mice was indifferent from WT mice at 7 dpi. This, combined with the severely blunted levels of CCL3 in the lungs of TLR7 KO mice at the same timepoint, supports an overall reduced LRT pathology in TLR7-deficient mice, further suggesting the partial localization of the inflammatory response to the URT in those mice.

A deeper analysis of the Th1/Th2 immune landscape in our model shed further light on the potential mechanisms underlying the manifestation of chronic RSV disease, particularly given the known contribution of Th2 dominant immune activation observed in infant cases of severe RSV infection, potentiating asthma and allergy sensitization (9, 51, 52). Notably, infection of neonatal mice with RSV enhanced the airway hyperreactivity response to subsequent allergen challenge, with higher eosinophil, macrophage and CD4+ T cell accumulation and elevated IL-4, IL-5 and IL-13 cytokines (53). In our study, the relative proportions of M2 compared to M1 macrophages in the upper airways of WT mice was highest during early infection and, in concordance with eosinophilia and expression of IL-13 in the lung, suggested a Th2 dominant phenotype that was absent in TLR7 KO mice. In WT mice at day 7, we observed a dominant population of M1 macrophages, which coincided with a Th1-skewed phenotype in the lung accompanied by high IFNγ:IL-13 ratios that persisted to day 42. The IFNγ:IL-4 ratios in the lung show that the loss of TLR7 appeared to initiate a stronger Th1 response during early infection, although there was no difference at 7 dpi. Neither mouse genotype exhibited significantly altered IL-5 levels, which is often associated with severe eosinophilic asthma, particularly following RSV infection (54). This suggests that the airway eosinophilia we observed in WT mice was probably driven by IL-13 and IL-4 resulting in a less overall severe eosinophilic pathology to infection, and suggesting other cell types contributed to the immunopathology observed (55). Interestingly, RSV-induced airway hyperreactivity has been reported to occur independently of increases in IL-13 (56). This suggests that the mechanism behind chronic manifestations of RSV disease, at least in older cohorts, might stem from hyperinflammatory reactions of prolonged Th1 responses, and not an exacerbated Th2 inflammatory response. Overall, this analysis implies that RSV infection induces a TLR7-mediated imbalance of Th1/Th2 responses, likely driving an early Th2 skew in the URT followed by a Th1 skew in the LRT later in the infection and pathogenesis process. Loss of TLR7 appeared to stabilize this Th1/Th2 balance, which improved acute lung pathology and had lasting effects on chronic disease.

We observed a population of pDCs retained in the airways up to 42 dpi, implying antigen presentation was occurring several weeks following acute responses to infection. Consistent with other studies (31, 32, 57, 58), low levels of RSV mRNA persisted in the lungs (and nasal tissue) at 42 dpi, but there were equivalent, if not higher amounts in TLR7 KO mice; which correlated with significantly less pDCs, patrolling monocytes and cytotoxic T cells. Thus, while TLR7 modestly influenced viral load in the URT, our data imply TLR7 is an unlikely contributor to RSV viral persistence. It remains unclear whether the mRNA transcripts detected at day 42 and thereafter represent the presence of virus capable of replication and potential reactivation. The use of standard plaque assays employed to detect replicating RSV from lung tissue is limited to acute infection protocols as the sensitivity falls below the detection limit of the assay after 7 dpi RSV (39). However, Schwarze et al. (32) were able to amplify the G, F, NS1, NS2 and M2 genes by RT-PCR from lungs of RSV infected mice up to 60 dpi, suggesting that the RSV genome can persist and contains the intact regions required for cell fusion and replication rather than being random viral fragments. Persisting viral RNA and presentation could indeed stimulate chronic activation of lingering CD8+ T cells, leading to T cell exhaustion and even airway immunopathology (59), although pre-existing RSV-specific “memory” CD8+ T cells within the airways of healthy individuals, correlated with reduced disease severity following RSV challenge (60). Importantly, we identified that mice experiencing the greatest airway and lung resistance upon MCh challenge (RSV-infected WT mice after 42 dpi) had the highest CD8+:CD4+ T cells ratios in the airways. This correlation existed in both mouse genotypes although TLR7 KO mice displayed a lower CD8+:CD4+ T cell ratio and minimal airway hyperreactivity. This is consistent with a study that reported infants who developed respiratory distress or were infected with RSV or human metapneumovirus had a skewed CD8+:CD4+ T cell ratio and increased IL-6 levels in their airways (61). The degree of the CD8+ T cell response also correlated with disease severity. Our results imply a potential relationship between RSV-induced airway hyperreactivity and CD8+ T cell persistence in the airways, and that the magnitude of this persistence is likely exacerbated by the TLR7-mediated hyperinflammatory responses during acute infection. Future experiments suppressing pulmonary T cell recruitment will provide important insight into the chronic consequences of T cell-mediated immunopathology during RSV infection.

Using TLR7 KO mice, Lukacs et al. (29) reported IL-17A-dependent mucus production during early RSV infection (3 dpi) that was increased in TLR7-deficient mice. IL-17A levels were similarly higher in TLR7 KO mice in our study, although we observed an overall reduction of immune infiltration in the BALF across 4 and 7 dpi. Conversely, we found that expression of mucin genes was lower in TLR7 KO mice implying excess mucus secretion in response to infection occurred in the presence of TLR7. This complemented our observations of LRT pathology. Consistent with Lukacs et al. (29), there was no difference in T cell numbers in the lungs, although we did observe slightly less DC subsets, NK cells and monocytes, overall emphasizing that the alteration in the immune response to RSV infection in TLR7 KO mice primarily impacted airway pathology in both models. IL-17A levels have been implicated in autoimmune disease pathogenesis and contribute to impaired lung function caused by respiratory viruses like RSV (62, 63). It is possible that the modulation of the IL-17A signal following its early Th17 response was better regulated in TLR7 KO mice as IL-6, an IL-17A-stimulating factor (64), was more effectively resolved and type I IFN, which is an IL-17A suppressing factor (65), was initially higher. Furthermore, Th17 cells reportedly influenced CD8+ cytotoxic T cell function in the airways in a model of chronic obstructive pulmonary disease (COPD) (66, 67) and impacted their virus-specific functions (68). Different strains of RSV A elicit slightly different immune responses and pulmonary pathophysiology in mice (69) and may account for the differences between our study and Lukacs et al. (29). We used RSV A Long strain, which induces a stronger type I IFN response in both epithelial and immune cells than RSV A2 strain (70). A greater type I IFN response, as observed in our RSV-infected TLR7 KO mice at 4 dpi, improves the rate of viral clearance and reduces pathology (71), thus infection with RSV A Long may represent a less severe, acute model in comparison to some other viral subtypes. Host responses to this strain also appear to favor Th1 immunity as Th1 but not Th2 cytokines were preferentially upregulated following RSV A Long infection compared to the line 19 strain, and this reduced the mucus-related pathology (72, 73). There is also evidence to suggest that the A2 strain can evade TLR recognition (70). Lukacs et al. (29) did not report IFN levels in the lungs, but observed no difference in IFN expression when DCs were infected with RSV *ex vivo*.

Host inflammatory responses to infection need to appropriately complement the acute antiviral mechanisms that clear the virus and cause minimal tissue damage and immunopathology. Our study demonstrates that TLR7 promotes a modest antiviral effect in the URT, but potentiates prolonged airway inflammation in the LRT, establishing a persistent inflammatory phenotype that drives chronic airway hyperreactivity. Therefore, the specific modulation of TLR7 during acute RSV infection may prevent individuals from developing severe RSV-induced pathology. For the first time, we can consider the novel approach of confining viral infections to the URT in order to prevent the serious inflammatory and pathophysiological ramifications of viral penetration into the LRT.

## 4 Materials and methods

### 4.1 Mice

Female C57BL/6J mice were obtained from the Animal Resources Centre (Western Australia, Australia). Homozygous TLR7 knockout mice (B6.129S1-Tlr7^tm1Flv^/J) were obtained from The Jackson Laboratory (Maine, USA) and bred in-house at the RMIT University animal research facility (Bundoora, Australia). Mice were housed in a 12 h light/12 h dark cycle with food and water.

### 4.2 Preparation of RSV stocks

Human respiratory syncytial virus (RSV A Long strain) was kindly provided by Prof. Patrick Reading (Department of Immunology and Microbiology, The Peter Doherty Institute for Infection and Immunity, University of Melbourne). RSV stocks were propagated on HEp-2 cells by incubating infected cells at 37°C, 5% CO2 for 3–4 days until at least an 80% cytopathic effect was observed. Culture medium was then removed, cell debris pelleted, and supernatant filter sterilized with a 0.22 µm Filter-Stericup. Virus was precipitated using poly-ethylene glycol (PEG) 6000 (Merck) and NaCl to a final concentration of 10% by incubating on ice for 2 h with gentle shaking. Virus was then pelleted and resuspended in phosphate buffered saline (PBS, Sigma, USA) + 1% fetal bovine serum (FBS; Sigma, USA). Viral titres were determined by plaque assay using HEp-2 cells.

### 4.3 RSV infection

For *in vivo* infection, 8–14-week-old female mice were anaesthetized by isoflurane inhalation and inoculated intranasally with 5 × 10^6^ (acute infection) or 2 × 10^7^ (long-term infection) plaque forming units (PFU) of RSV-A or PBS for controls in a 35 µL volume. Data were compared against PBS mice to control for any baseline anti-inflammatory effects from the anesthetic procedure. The greater viral inoculum administered for the long-term infection experiments was based on previous studies assessing RSV-induced chronic defects in lung function (31, 58). Mice were weighed and monitored daily. Mice were euthanized by injection (i.p) of a mixture of ketamine (180 mg/kg) and xylazine (32 mg/kg) at experimental endpoints. All animal experiments were conducted according to approval obtained from the RMIT University Animal Ethics Committee (Ethics number 23328) and in compliance with the guidelines of the National Health and Medical Research Council of Australia on animal experimentation.

For *ex vivo* infection, primary alveolar macrophages were extracted from bronchoalveolar lavage (BAL) and grown in Dulbecco’s Modified Eagle’s Medium (DMEM; Thermofisher, USA) containing 4.5 g/L of glucose, 110 mg of sodium pyruvate and 10% FBS in flat 96-well plates (10^5^ cells per well) and allowed to adhere for 3–4 h prior to infection. Media was then replaced, and cells infected with RSV at a multiplicity of infection (MOI) of 1 and incubated at 37°C, 5% CO2 for 24 h before direct cell lysis for RNA extraction.

### 4.4 Airways inflammation

To assess airway inflammation, bronchoalveolar lavage fluid (BALF) was isolated by an incision of the lower jaw to the top of the rib cage to expose the salivary glands, which were separated to expose the top of the trachea. A small incision on the trachea was made and a sheathed 21-Gauge needle was inserted into the lumen. The lung was then lavaged with 300–400 µL aliquots of PBS repeatedly with gentle massaging of the chest with each aspirate collected until a volume of 1 mL was collected. The total number of live cells in the BALF was determined via Acridine Orange staining (Thermofisher) and counted using a hemocytometer. Differential staining of BAL was performed as previously described (74) and scored using standard morphological criteria counting at least 500 cells/slide from random fields.

### 4.5 Histology analysis of lungs

The left lung was dissected from mice and immersed in neutral buffered formalin (10%). After fixation, the lung tissue was processed, embedded in paraffin wax, and longitudinal 4 μm sections cut and stained with hematoxylin and eosin (H&E). Slides were scanned by light microscopy using the Olympus VS-120 Slide Scanner (Olympus Life Sciences, VIC, Australia). Histology was analyzed blindly by two independent assessors using Olympus OlyVIA imaging software. Each sample was scored from 0–5 for each individual mouse (higher numbers indicate increased severity). At least five random fields from each lung section were assessed for alveolar inflammation of the parenchyma (alveolitis), inflammation around the bronchiolar airway wall (peribronchiolar inflammation), and total inflammatory cell infiltration. Alveolitis was defined based on the regularity and branching of the alveoli as well as the density of cells within the alveolar spaces (interstitium). Peribronchiolar inflammation was characterized by immune cell infiltration into the alveolar wall around the bronchioles. Analysis of bronchioles were limited to airways of 100- to 350-µm luminal diameter. The degree of inflammatory cellular infiltrate was taken by observing the density of cells throughout the entire lung section.

### 4.6 Gene expression analysis by reverse-transcriptase quantitative PCR

Lungs and nasal tissue were harvested for RNA extraction using the RNeasy Mini kit (Qiagen, USA), as per manufacturer’s instructions. RNA sample concentration and quality were measured using the Nanodrop one Spectrophotometer (Thermofisher). The cDNA synthesis was performed on 1–2 μg of total RNA using the High-Capacity cDNA Reverse Transcription Kit (Applied Biosystems, CA, USA) according to the following settings: 25°C for 10 min, 37°C for 120 min, 85°C for 5 min. Quantitative polymerase chain reaction was carried out using the TaqMan Fast Advanced Master Mix (Thermofisher) and analyzed on the QuantStudio 7 Flex Real-Time PCR system (Thermofisher). PCR primers used in this study were included in the Assay on-Demand Gene Expression Assay Mix (Thermofisher). Additionally, RSV titres were measured using custom designed forward and reverse oligonucleotides for the F gene: 5′-TTGGATCTGCAATCGCCA-3′, 5′-CTTTTGATCTTGTTCACTTCTCCTTCT-3′ using Fast SYBR Green PCR Master Mix (Thermofisher). The following program settings were used for amplification: 50°C for 2 min, 95°C for 2 min, then 40 cycles of 95°C for 1 s and 60°C for 20 s. The quantitative values were obtained from the average threshold cycle (Ct) number of each sample run in triplicate and gene expression analysis performed using the comparative Ct method. Target gene expression was normalized against RPS18 mRNA expression for each sample and expressed relative to the indicated control. Mean Ct numbers for RPS18 for each experimental group are shown in **Supplementary T** **able S1**.

### 4.7 Cytokine protein levels by enzyme-linked immunosorbent assay

Enzyme-linked immunosorbent assays (ELISA) were performed to quantitate cytokine protein levels from whole lung tissue (IL-1β), BALF (secreted IFNγ) or cell culture supernatants (secreted IFNβ or IL-6). Individual mouse IL-1 beta/IL-1F2, IFN-gamma, IFN-beta or IL-6 DuoSet ELISA kits were used (R&D System, MN, USA). Lung lysates were generated by resuspending tissue in RIPA buffer containing protease inhibitor and passing tissue through a 25-Gauge needle multiple times. Protein concentrations were determined from cleared lysates using the Pierce BCA Protein Assay kit (ThermoFisher) according to manufacturer’s instructions. One hundred micrograms of protein was added in duplicate to pre-coated 96-well plate and incubations performed according to manufacturer’s instructions. To measure secreted proteins, 100 µl of BALF or cell culture supernatant was added in duplicate and processed in the same manner. The 96-well plate was read on the CLARIOstar (BMG) at a wavelength of 450 nm. Cytokine titres in the samples were determined by plotting the optical densities, using a four-parameter fit for the standard curve and expressed in pg/mL.

### 4.8 Immunophenotyping by flow cytometry

Whole lung was finely minced using scissors and then enzymatically digested using 1% Liberase (Sigma) for 45 min at 37°C shaking at 700 rpm. Tissues were homogenized then single cell suspensions prepared by straining through a 40 µm strainer. After lysing the red blood cells with ACK lysis buffer, cells were stained with cocktail mixtures of fluorescent-labelled anti-mouse antibodies diluted in FACS buffer (PBS + 2.5% FBS) for 30 min on ice. The following Biolegend antibodies were used (unless stated otherwise): CD45-PerCP (30-F11), CD3e-APC (145-2C11; BD Pharmingen), CD4-BV605 (RM4-5), CD8a-PE-Cy7 (53-6.7), NK1.1-FITC (PK136), CD11b-BV421 (M1/70), CD11c-PE-Cy7 (N418; eBioscience), PDCA-1-PE (JF05-1C2.4.1; Miltenyi Biotec), MHC-II-APC (M5/114.15.2), Ly6C-FITC (HK1.4), Ly6G-APC-Cy7 (1A8), F4/80-PE (BM8; eBiocience), iNOS-FITC (6; BD Transductions Laboratories) and CD206-PE-Cy7 (MMR; eBioscience). CD16/32 (2.4G2) and LIVE/DEAD Fixable Aqua Dead Cell Stain Kit (Invitrogen) were contained within each antibody cocktail mixture to block of Fc-mediated adherence of the antibodies and to exclude dead cells, respectively. Samples were processed on a BD LSRFortessaTM X-20 flow cytometry analyzer with DIVA software (Becton Dickinson Bioscience, USA) and data analyzed using FlowJo software (Tree Star, Inc.). Cells were analyzed as a percentage of the CD45-positive (live cells) and lung cells expressed in absolute numbers per 10,000 counting beads and amount of tissue (g) processed. BAL cells were expressed in absolute numbers relative to live cell counts. A representative gating strategy is shown in **Supplementary Figure S2**.

### 4.9 Airway hyperresponsiveness and lung function

Mice were anesthetized by ketamine (80 mg/kg) and xylazine (16 mg/kg) after which tracheotomy was performed by inserting an 18-Gauge canular into the trachea. Airway reactivity in response to increasing doses of nebulized methacholine (MCh; Sigma) or PBS was measured *in vivo* using the Flexivent FX1 (SCIREQ®, QC, Canada). Forced oscillation technique was used to measure the pressure, flow and volume responses to measure total airway resistance (Rrs), Newtonian resistance (Rn; representing resistance of the central/conducting airways) and tissue dampening (G; representing resistance of the small airways and alveolar space).

### 4.10 Statistical analysis

All data are expressed as the mean ± SEM. All comparisons were performed using GraphPad Prism (GraphPad Software Version 8.2, USA) and performed by two-way ANOVA followed by Tukey’s or Sidak’s *post-hoc* tests for multiple comparison (stated in figure legends). Statistical significance was considered at p<0.05.

## Data availability statement

The original contributions presented in the study are included in the article/**Supplementary Material**. Further inquiries can be directed to the corresponding author.

## Ethics statement

The animal study was approved by RMIT University Animal Ethics Committee. The study was conducted in accordance with the local legislation and institutional requirements.

## Author contributions

MM, SL, FL, MC-S, GT, JE, OO performed the experiments. MM and SS drafted the manuscript. All authors provided intellectual input and edited the manuscript. MM, JO’L, DB and SS conceptualized and designed the study. RB, SH, JL, HW, SB, DB, JO’L and SS contributed to the experimental design of this research project. DB, JO’L and SS obtained the funding for the research. SS supervised and managed the overall study. All authors contributed to the article and approved the submitted version.
