# Supplementary material for: TLR7 promotes chronic airway disease in RSV-infected mice
Source: Front Immunol. 2023 Sep 14;14:1240552. doi: 10.3389/fimmu.2023.1240552 (PMC10545951; doi:10.3389/fimmu.2023.1240552)
Supplement: Supplementary file 1 [file DataSheet_1.pdf]

RMIT Classification: Trusted

Supplementary Material

TLR7 promotes chronic airway disease in RSV-infected mice

Mark A. Miles[1], Stella Liong[1], Felicia Liong[1], Madison Coward-Smith[1], Gemma S. Trollope[1], Osezua Oseghale[1], Jonathan R. Erlich[1], Robert D. Brooks[2], Jessica M. Logan[2], Shane Hickey[2], Hao Wang[1], Steven Bozinovski[1], John J. O'Leary[3,4,5], Doug A. Brooks[2,3] and Stavros Selemidis[1]*

[1] Centre for Respiratory Science and Health, School of Health and Biomedical Sciences, RMIT University, Bundoora, Victoria, 3083, Australia.

[2]Clinical and Health Sciences, University of South Australia, Adelaide, 5001, Australia.

[3]Discipline of Histopathology, School of Medicine, Trinity Translational Medicine Institute (TTMI), Trinity College Dublin, Ireland.

[4]Sir Patrick Dun's Laboratory, Central Pathology Laboratory, St James's Hospital, Dublin, Ireland.

[5]CERVIVA research consortium, Trinity College Dublin, Ireland.

*** Correspondence:**
Stavros Selemidis
stavros.selemidis@rmit.edu.au

RMIT Classification: Trusted

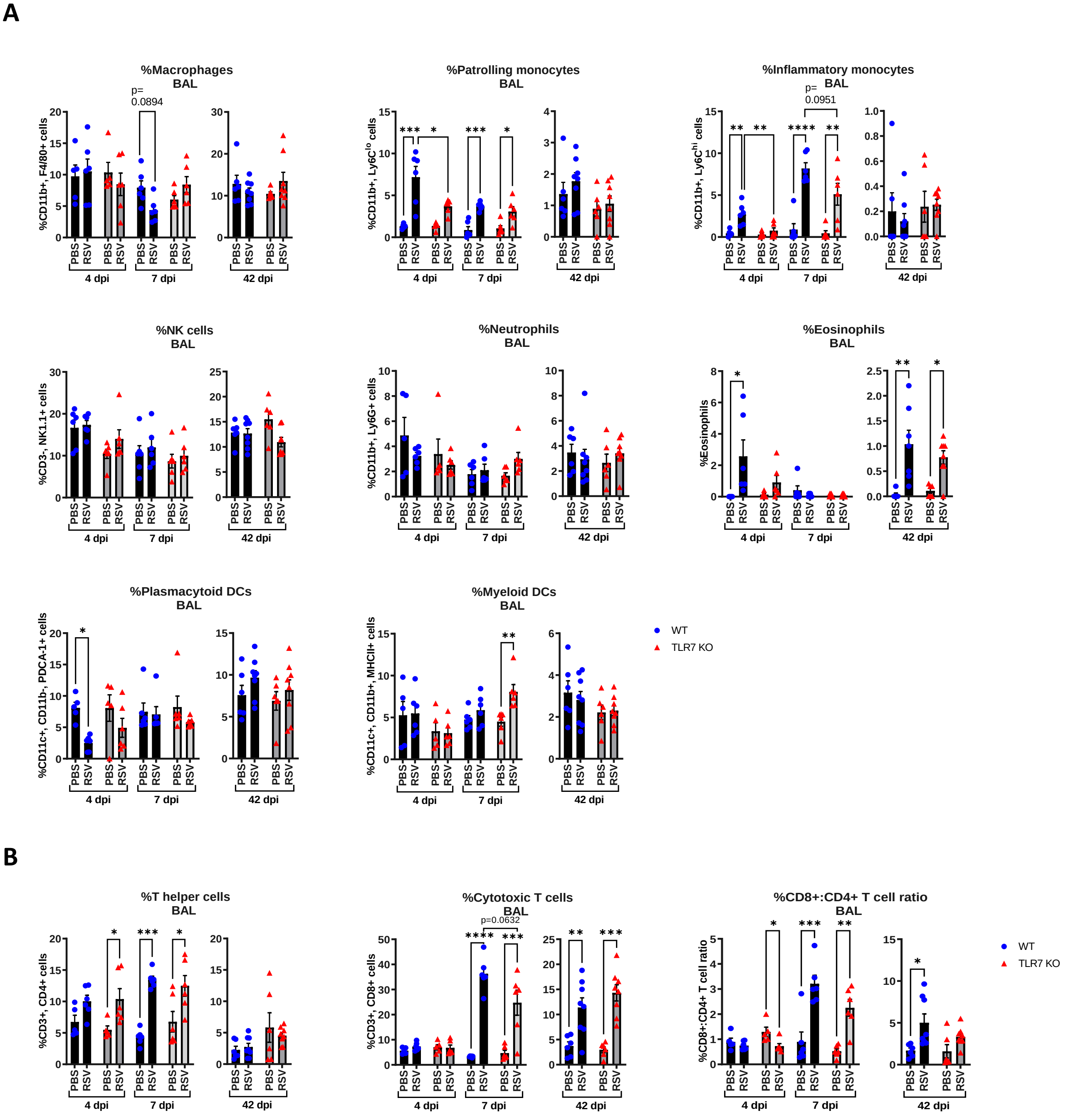

Supplementary Figure S1. Airway immune composition is not dramatically altered in TLR7 KO mice following RSV infection. WT C57BL/6 or TLR7 KO mice were infected with RSV (0.5-$2x10^7$ PFUs) or PBS via intranasal administration and analysis performed after 4, 7 or 42 dpi. The percentage of immune cell populations in the airways collected in the BAL fluid was determined by flow cytometry. (A) Innate immune cell types: interstitial macrophages ($CD11b^+$ $F4/80^+$), patrolling monocytes ($CD11b^+$ $Ly6C^{lo}$), inflammatory monocytes ($CD11b^+$ $Ly6C^{hi}$), NK cells ($CD3^-$ $NK1.1^+$), neutrophils ($CD11b^+$ $Ly6G^+$), plasmacytoid DCs ($CD11c^+$ $CD11b^-$ PDCA-1^+) and myeloid DCs ($CD11c^+$ $CD11b^+$ $MHCII^+$). Numbers of differentially stained eosinophils was determined by counting 500 cells from random fields by standard morphological criteria relative to the total number of isolated cells. (B) Adaptive T cell subtypes: T helper ($CD3^+$ $CD4^+$) and cytotoxic T cells ($CD3^+$ $CD8^+$). Cell populations were measured as percentage of $CD45^+$ cells. Data are expressed as mean ± SEM, n = 5-8 mice per experimental group from two independent experiments. Statistical analysis was conducted using two-way ANOVA test followed by Tukey's post hoc test for multiple comparison test (*$p < 0.05$, **$p < 0.01$, ***$p<0.001$, ****$p<0.0001$).

RMIT Classification: Trusted

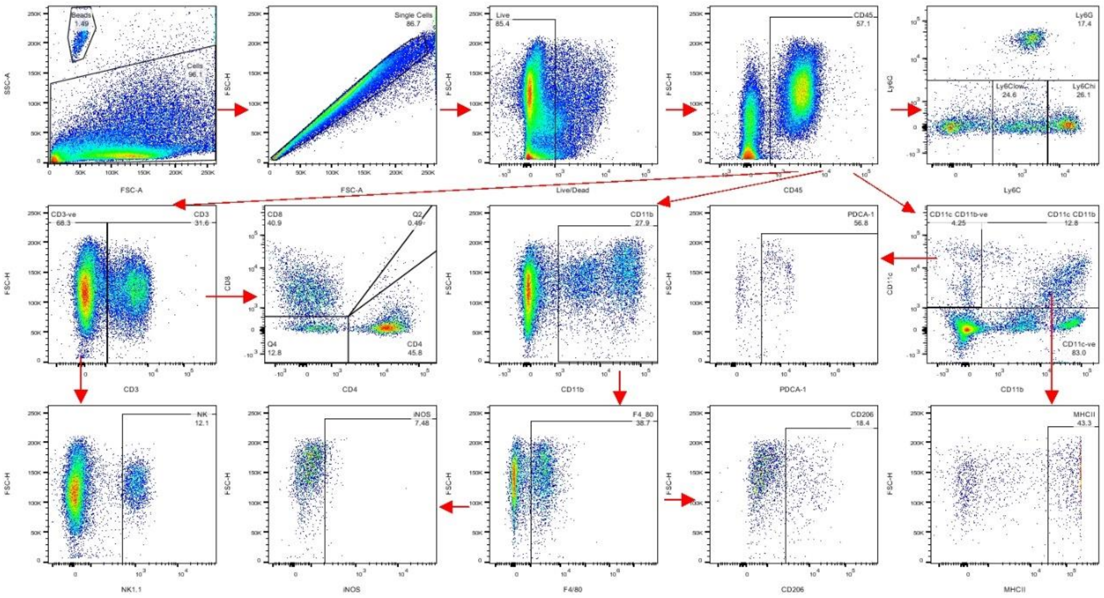

Supplementary Figure S2. Flow cytometry gating strategy. Lymphocytes and myeloid cells were gated as CD3$^+$ or CD11b$^+$ respectively from CD45$^+$ leukocytes. CD3$^+$ T cells were further divided into subsets T helper (CD4$^+$) and cytotoxic (CD8$^+$) T cells. NK cells (NK1.1$^+$) were gated from CD3$^-$ populations. Patrolling Ly6C^{lo}, pro-inflammatory Ly6C^{hi} monocytes and inflammatory Ly6G$^+$ neutrophils were identified within CD11b$^+$ myeloid cells. F4/80$^+$ macrophages were identified from CD11b$^+$ cells then further divided into subsets M1 proinflammatory (iNOS$^+$) and M2 anti-inflammatory (CD206$^+$). Dendritic cell subsets were gated from CD11b$^+$/CD11c$^-$ populations (plasmacytoid, PDCA-1$^+$) or CD11b$^+$/CD11c$^+$ populations (myeloid, MHCII$^+$).

Supplementary Table S1. RSV Fusion gene and RPS18 housekeeping cycle threshold (Ct) values of lung and nasal tissue from PBS control and infected mice from 4, 7 and 42 dpi study timepoints.

Study endpoint	Genotype	Infection status	Lung tissue [b]			Nasal tissue [c]		
			Ct RSV	Ct RPS18	ΔCt (x 10^4) [a]	Ct RSV	Ct RPS18	ΔCt (x 10^4) [a]
4 dpi	WT	PBS	31.21	21.04	10.23	38.47	22.73	0.15
		RSV	18.69	20.98	55679.65	27.95	24.28	831.10
	TLR7 KO	PBS	28.88	21.03	63.29	37.45	24.45	1.56
		RSV	18.7	21.04	55866.14	29.83	25.28	705.97
7 dpi	WT	PBS	33.73	21.13	2.97	38.78	26.64	3.12
		RSV	24.31	21.23	1454.06	31.42	23.31	41.53
	TLR7 KO	PBS	35.04	21.27	1.28	36.46	25.06	4.21
		RSV	24.35	20.97	1309.66	29.99	23.36	77.94
42 dpi	WT	PBS	36.5	21.46	0.35	37.16	22.26	0.48
		RSV	33.62	20.92	2.22	35.77	23.47	5.35
	TLR7 KO	PBS	36.36	21.51	0.41	38.88	22.63	0.25
		RSV	33.22	21.67	3.97	37.34	24.43	4.18

[a] Cycle threshold values for RSV Fusion gene amplification relative to RPS18 housekeeping

[b] *n* = *6-8* in both PBS controls and RSV infected groups

[c] *n* = *5-8* in both PBS controls and RSV infected groups
